# Improving stability of atlantoaxial fusion: a biomechanical study

**DOI:** 10.1007/s00590-022-03465-y

**Published:** 2022-12-22

**Authors:** Adrian Cavalcanti Kußmaul, Titus Kühlein, Axel Greiner, Sandy Walter, Christopher A. Becker, Manuel Kistler, Bianka Rubenbauer, Sebastian Andreß, Wolfgang Böcker, Jan Bruder

**Affiliations:** Department of Orthopaedics and Trauma Surgery, Musculoskeletal University Center Munich (MUM), University Hospital, LMU Munich, Marchioninistr. 15, 81377 Munich, Germany

**Keywords:** Biomechanics, Cervical spondylodesis, Cervical fracture, Atlantoaxial instability, Odontoid fracture

## Abstract

**Purpose:**

The incidence of atlanto-axial injuries is continuously increasing and often requires surgical treatment. Recently, Harati developed a new procedure combining polyaxial transarticular screws with polyaxial atlas massae lateralis screws via a rod system with promising clinical results, yet biomechanical data is lacking. This biomechanical study consequently aims to evaluate the properties of the Harati technique.

**Methods:**

Two groups, each consisting of 7 cervical vertebral segments (C1/2), were formed and provided with a dens axis type 2 fracture according to Alonzo. One group was treated with the Harms and the other with the Harati technique. The specimen was loaded via a lever arm to simulate extension, flexion, lateral flexion and rotation. For statistical analysis, dislocation (°) was measured and compared.

**Results:**

For extension and flexion, the Harati technique displayed a mean dislocation of 4.12° ± 2.36° and the Harms technique of 8.48° ± 1.49° (*p* < 0.01). For lateral flexion, the dislocation was 0.57° ± 0.30° for the Harati and 1.19° ± 0.25° for the Harms group (*p* < 0.01). The mean dislocation for rotation was 1.09° ± 0.48° for the Harati and 2.10° ± 0.31° for the Harms group (*p* < 0.01). No implant failure occurred.

**Conclusion:**

This study found a significant increase in biomechanical stability of the Harati technique when compared to the technique by Harms et al. Consequently, this novel technique can be regarded as a promising alternative for the treatment of atlanto-axial instabilities.

## Introduction

The incidence of injuries of the upper cervical spine is continuously increasing [1], especially with regard to the current demographic change and the concomitant rise of compromised bone tissue [2].

Considering the trauma mechanism, elderly patients usually present with a base-near odontoid fracture, that according to Anderson and D’Alonzo is defined as a type II fracture and generally results from low-impact or inadequate trauma [3, 4].

In general, axis fractures make up one-third of all fractures of the cervical spine with approximately half of them compromising the dens axis [1, 4]. These injuries are oftentimes associated with a high morbidity and mortality [2]. Due to the proximity of the cervico-medullary junction and the extensive mobility of the cranial-cervical junction, instabilities located in this area bear the risk of life threatening neurological damage and often require surgical intervention [5, 6]. Here, a spondylodesis provides rapid stabilization both protecting the spinal cord and minimalizing potential neurological complications [7].

Over the past decades, many techniques have evolved to treat atlanto-axial instabilities. While anterior screw osteosynthesis is commonly used in younger patients with minor instability and little dislocation as well as for spondylodesis in older patients in combination with transarticular C1–C2 screws [4], cases with severe instabilities and significant dislocation impeding anterior screw placement usually require dorsal stabilization [4].

For example, Harms et al. connected atlas massae lateralis screws to axis isthmus screws via a rod system, resulting in a rigid spondylodesis based on the principle of an internal fixation [8]. Therefore, this technique is characterized by a facilitated implantation with neither the need for continuous fluoroscopy to secure the vertebral artery nor requiring a preoperative reduction maneuver [9]. In addition, the joint surface between C1 and C2 remains unaffected, potentially maintaining joint mobility after removal [8]. Yet, a potential disadvantage of this technique lies in the lower stability of both a two-point fixation and short screws. Furthermore, the pivot point is shifted outside the vertebra and thus burdens the screw rod connection [9].

Furthermore, Magerl et al. described a posterior transarticular C1–C2 osteosynthesis [10]. Here, the implantation of a transarticular screw is biomechanically stable, yet its implantation is challenging and due to the required steep insertion angle not feasible in patients with obesity or scoliosis [11]. Likewise, an anomalous course of the vertebral artery, which occurs in about 20% of the population, is a contraindication for the transarticular screw implantation [10].

Recently, Harati et al. performed a case study in which the authors first combined the transarticular screw described by Magerl with the Harm’s screw rod system with promising results as they found no intra- or postoperative complications and reported no screw loosening or dislocation after a follow-up of 36 months, ultimately presenting their technique as a safe and effective method for the stabilization of posttraumatic atlanto axial instabilities [7, 12]

Even though fusion rates for dorsal stabilization have found to be higher than for anterior osteosynthesis techniques [2, 4, 13], non-union rates despite surgical treatment are still relevantly high, resulting in an ongoing debate about the different techniques and their implantation [1].

Especially regarding the lack of biomechanical studies investigating the properties of the combined technique by Harati et al., it can currently hardly be compared with other, more established posterior spondylodesis procedures. Yet, this osteosynthesis method seems to be a promising alternative for the treatment of atlanto-axial instabilities, ultimately demanding an in vitro biomechanical study to comprehend and validate its biomechanical properties.

Consequently, this study aims to investigate and compare the biomechanical properties of the Harati technique.

## Material and methods

This study assumes that the Harati technique improves biomechanical stability compared to the Harms technique. Therefore, 14 synthetic cervical columns (Spine, Cervical 1351, Sawbone® Pacific Research Laboratories, Vashon, WA, USA) were randomly divided into two groups. The C1–C2 segments were isolated, maintaining the anterior longitudinal ligament (ALL) and posterior longitudinal ligament (PLL) in between C1 and C2 for transitional stability during implant placement.

**Fig. 1:**
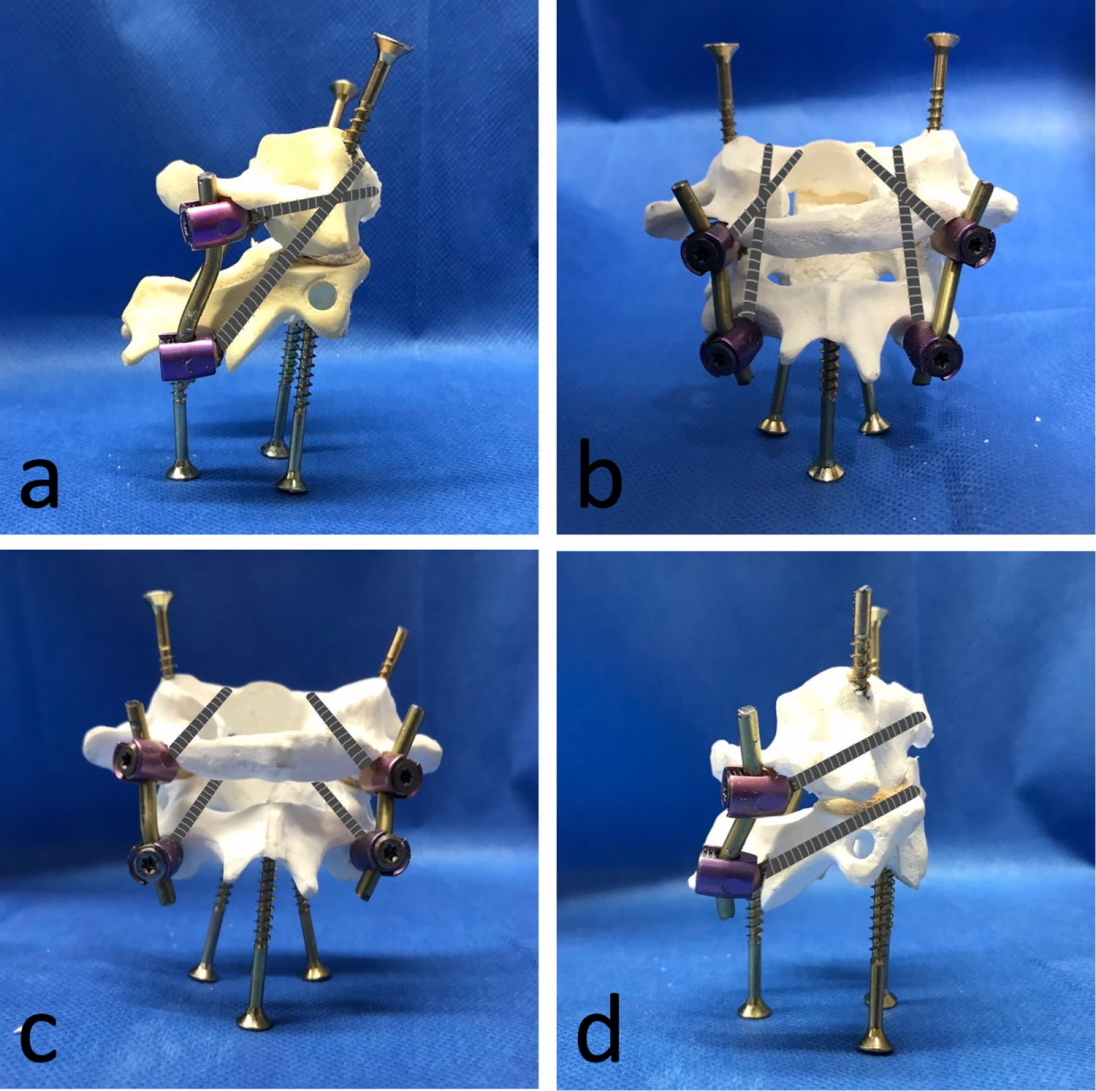
Visualized screw position of the osteosynthesis technique by Harati et al. (**a**, **b**) and Harms et al. (**c**, **d**)

A type 2 dens axis fracture according to Anderson and D’Alonzo was created by transecting the dens axis at the base [3]. The ALL and PLL were removed to create a maximum instability and to ensure comparable conditions as their tension and fixation potentially differ between individual specimens. Each vertebra was provided with additional screws placed both cranially and caudally in the vertebral bodies to generate a bigger surface and to optimize rigid fixation in the resin (see Fig. 1) [14]. No screw broke through the opposite corticalis and there was no interference with the osteosynthesis screws.

Each group was instrumented with one of the two procedures described below:

- *Harms technique (Harms)* Two atlas massae lateralis screws (DePuy Synthes, Spine SYMPHONY™ OCT System, length: 18 mm, width: 3.5 mm) were implanted into the atlas. Landmark for placement of the pilot hole was the middle of the junction of the C1 posterior arch and the midpoint of the posterior inferior part of the C1 lateral mass. The hole was drilled in posterior-anterior direction parallel to the plane of the C1 posterior arch. The polyaxial head of the screw was positioned above the posterior arch [8].
  Subsequently, two axis isthmus screws (DePuy Synthes, Spine SYMPHONY™ OCT System, length 24 mm, width 3.5 mm) were inserted into the axis. Again, a pilot hole was drilled into the cranial and medial part of the isthmus surface of C2 in a posterior-anterior direction, slightly convergent and cephalad [8]. The screws were then connected by a pre-bent rod and tightened with innies (see Fig. 1).
- *Harati technique (Harati)* Two axis isthmus screws were placed into C1 as described above. Additionally, two transarticular screws (DePuy Synthes, Spine SYMPHONY™ OCT System, length: 38 mm, width: 3.5 mm), as first described by Magerl et al., were inserted. The entry points lay in the lower part of the caudad articular process of C2. The drilling was orientated towards the upper crest of the isthmus and about 15° converted. The screws were then again connected by a pre-bent rod and tightened with innies [10] (see Fig. 1).

K-wires were used to ensure precise screw positioning and a drill of 2.5 mm diameter was used to pre-drill the holes.

**Fig. 2:**
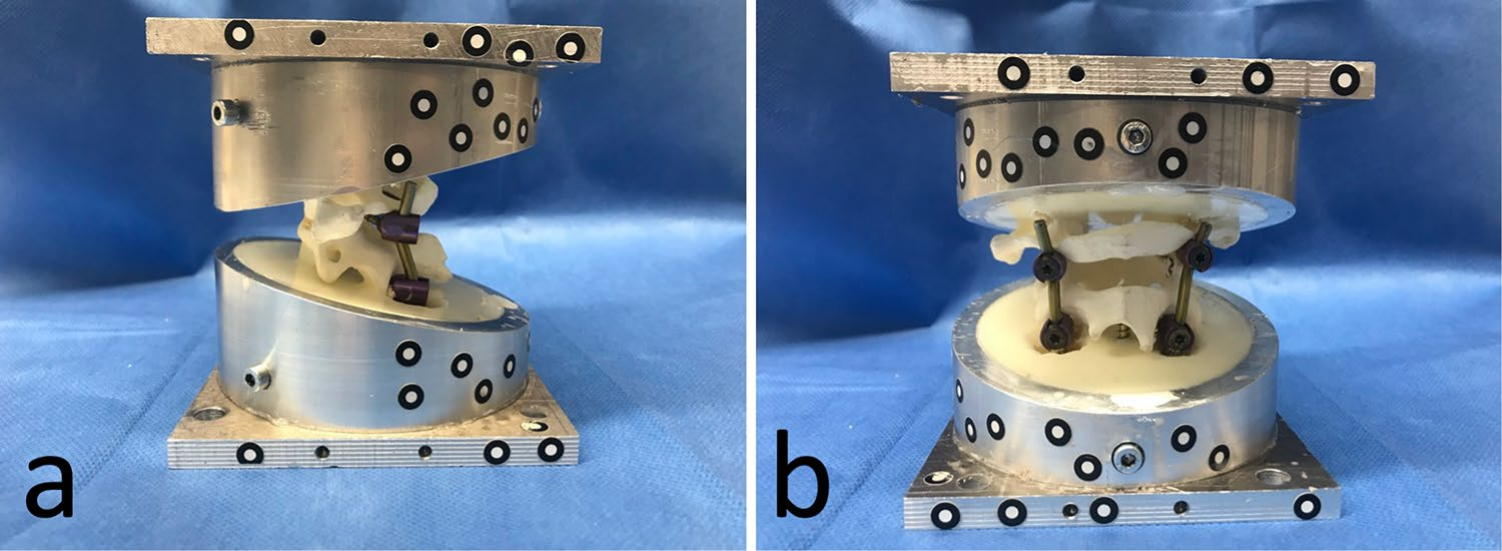
Embedded specimen from lateral (**a**) and posterior (**b**) view

**Fig. 3:**
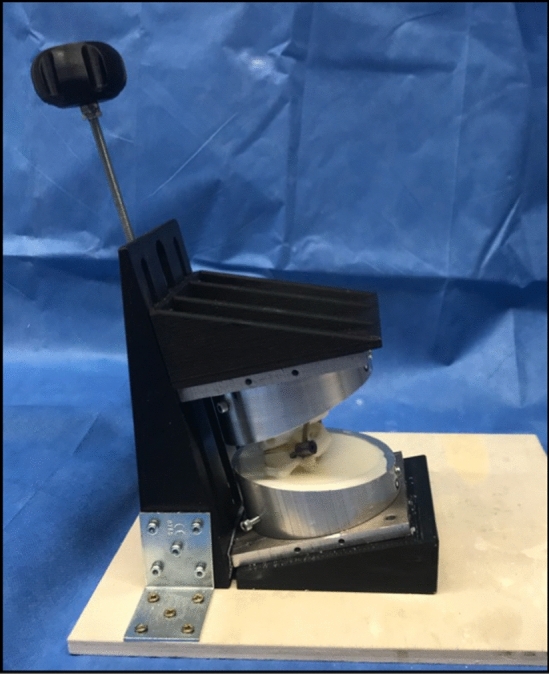
3D-printed casting guide to achieve a 13° casting line

The specimens were cast in specially designed pots filled with resin (RenCast® FC 52/53 Isocyanate/FC 53 Polyol, Huntsman Corporation®, Salt Lake City, UT, USA) using a casting guide (see Figs. 2, 3).

The upper edge of the pots was previously flattened by 13° to provide sufficient support while allowing enough space for implant placement. The casting guide was designed using CAD (program: Fusion 360® Autodesk, San Rafael, CA, USA) and 3D printed (3D-printer: Original Prusa i3MK3S + ®, Prusa Research, Prague, Czech Republique) to obtain an exact 13° casting line. To prevent the rods from being cast in, plasticine was fixed to their ends during casting.

**Fig. 4:**
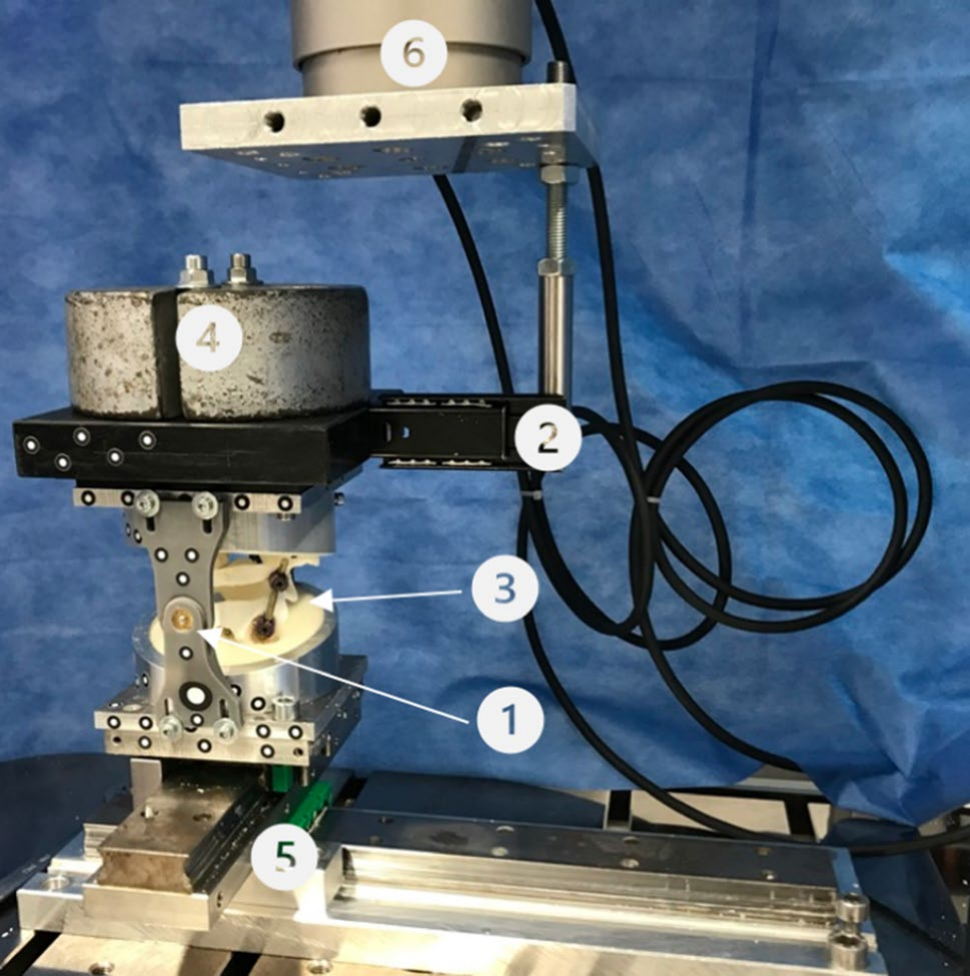
3D-printed supports with a central ball bearings-joint against rotational shear forces, 2 telescoping rail as lever arm, 3 sample, 4 initial load of 50 N, 5 *x*–*y*-table, 6 load cell of the testing machine

The lower pot was mounted on a free moving *x*–*y*-table allowing a rapid changeover. During testing, the table was blocked in both planes. The upper pot was brought into a torsional moment via a 15 cm long lever arm connected to the biomechanical testing machine (Instron e10000, Norwood, MA, USA). The set-up allowed a 90° rotation of the lever arm to simulate both flexion and extension in the sagittal plane as well as lateral flexion in the frontal plane. To absorb tensile forces during force transmission, the lever arm consisted of a ball bearing rail. In order to prevent any rotational shear forces, the pots were connected vertically to the tested axis via ball-bearing joints. During rotation, the torque moment was transmitted directly from the testing machine to the upper pot. To simulate the head weight, an initial weight of 5 kg was placed on the upper pot during testing in the sagittal and frontal plane. Adding the weight of the upper pot and resin, it accumulated to a total preload of 75 N. During rotation, the initial weight was applied directly via the testing machine (see Fig. 4).

All samples were loaded with a torque of ± 2 Nm in all 6 directions: In rotation via direct torque application of the testing machine and in the sagittal and frontal planes via ± 13.33 N tension and compression on the lever arm. A total of 15 cycles was performed, oscillating around 0 N. The maximum speed was set to 0.5°/s. All movements were measured by an optical sensor system (GOM Aramis 3D Camera 12 M, GOM GmbH, Braunschweig, Germany), and recorded with a frequency of 5 Hz. The biomechanical test set-up (see Fig. 4) and the testing protocol (see Table 1) were performed based on a study by Röhl et al. [14].

**Table 1:** Testing protocol

Step 1	Adjusting the loading to 0 N (baseline)	Extension/flexion
Step 2	Cyclic loading (15 cycles) between − 13,33 N and + 13,33 N	Extension/flexion
Step 3	Travelling back to the baseline position	Extension/flexion
*Change of experimental set-up to lateral bending*		
Step 4	Adjusting the loading to 0 N (baseline)	Lateral bending
Step 5	Cyclic loading (15 cycles) between − 13,33 N and + 13,33 N	Lateral bending
Step 6	Travelling back to the baseline position	Lateral bending
*Change of experimental set-up to rotation*		
Step 7	Adjusting the loading to 50 N (baseline)	Rotation
Step 8	Cyclic rotation (15 cycles) between − 2 N/m and + 2 N/m	Rotation
Step 9	Travelling back to the baseline position	Rotation

The mean dislocation (°) was calculated and, according to the distribution (Shapiro Wilk Test), means were compared using a *t* Test. A *p* < 0.05 was considered as statistically significant.

Statistical analysis was performed with IMB SPSS Statistics® version 28 (Armonk, NY, USA).

## Results

**Fig. 5:**
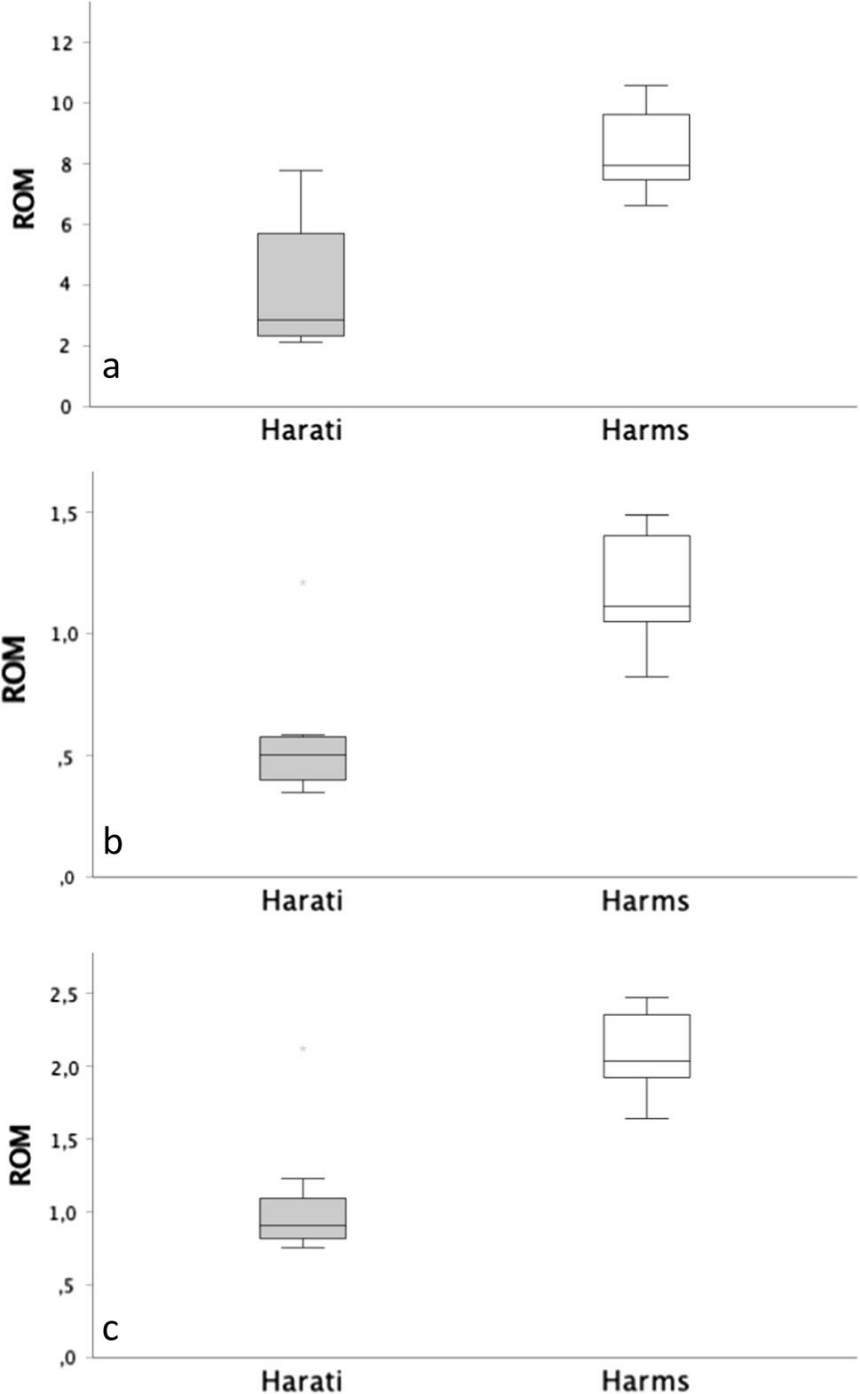
Comparison of the dislocation (range of motion (ROM) °) for flexion/extension (**a**), lateral flexion (**b**) and rotation (**c**)

Regarding movement in the frontal plane, the Harati group showed a mean dislocation of 4.12° ± 2.36°, ultimately displaying a significantly lower dislocation than the technique by Harms (8.48° ± 1.49°, *p* < 0.01) (see Table 2; Fig. 5). In the sagittal plane, this study also found a significantly less dislocation for the Harati (0.57° ± 0.3°) than for the Harms group (1.19° ± 0.25°, *p* < 0.01) (see Table 2; Fig. 5). Considering the results of the transversal plane, the Harati group again showed a significantly lower dislocation (1.09° ± 0.48°) than the Harms group (2.10° ± 0.31°) (*p* < 0.01) (see Table 2; Fig. 5).

**Table 2:** Dislocation of the osteosynthesis techniques (°)

		Mean dislocation	Level of significance
Extension/flexion	Harati group	4.12 ± 2.36	*p* < 0.01
	Harms group	8.48 ± 1.49	
Lateral flexion	Harati group	0.57 ± 0.30	*p* < 0.01
	Harms group	1.19 ± 0.25	
Rotation	Harati group	1.09 ± 0.48	*p* < 0.01
	Harms group	2.10 ± 0.31

## Discussions

The most important finding of this study was that the technique described by Harati et al. resulted in a significantly higher stability in all three motion planes than the technique by Harms et al. (*p* < 0.01). While both osteosynthesis methods sufficiently addressed the instability as all measured dislocations stayed below physiological mobility, the Harati technique almost reduced the dislocation compared to Harms by 51% for extension/flexion, 52% for lateral flexion and 48% for rotation regarding the individual dislocations in each plane.

Based on the foundation of its biomechanical stability, this novel osteosynthesis as a combination technique furthermore enables an unilateral implantation of a transarticular screw [7]. In detail, since an anomalous course of the vertebral artery, impeding the implantation of a transarticular screw, generally occurs unilaterally, this technique allows the implantation of an axis isthmus screw on the affected site while placing a transarticular screw contralaterally [7]. A further advantage of this technique lays in its potential to extend the technique to an occipito-cervical fusion in case of extensive injuries involving the atlanto-occipital joint or insufficient screw stability in compromised bone quality [15].

The increase of biomechanical stability by the Harati technique is a result of its three-point fixation. With regard to current literature, numerous studies have demonstrated that the sole use of a transarticular screw displays sufficient biomechanical stability but, when combined with an atlas massae lateralis screw, results in a three-point fixation system with an increased biomechanical stability [7, 16, 17].

These results are also consistent with the ones by Guo et al. in their biomechanical study, the authors compared five different dorsal stabilization procedures, including a dorsal cerclage by Gallie, a transarticular screw and cerclage by Gallie, a single transarticular screw, the combination of a transarticular screw and a laminar hook and a C1/C2 spondylodesis comparable to the one by Harms [16]. The authors proved that the three-point fixation technique combining transarticular screws with a laminar hook displayed significantly less dislocation in all planes compared to the two-point fixation by Harms [16]. In contrast to the Harati method, however, a bone graft needs to be harvested from the iliac crest, not only extending the surgical procedure but also increasing perioperative morbidity [16, 18]. Another study comparing different dorsal fusion procedures was performed by Sim et al., which was also able to confirm the biomechanical superiority of three-point fixations over two-point fixations with a significant difference of biomechanical stability in the sagittal and axial plane (*p* < 0.05) [9]. In detail, the three-point fixation used in this study consisted of the combination of a transarticular screw and a posterior wiring technique according to Gallie while the two-point fixation consisted of the combination of atlas massae lateralis screws with axis pedicle or laminae screws [9]. Yet, similar to the previously mentioned hook system according to Guo et al., a bone graft from the iliac crest is necessary [16], increasing the risk of pain, nerve and vascular injury, peritoneal perforation, sacroiliac joint instability, fractures and herniation of abdominal structures through defects in the ilium [18].

Regarding limitations, this study used synthetic cervical bone models with limited transferability to real life conditions. However, synthetic models are excellent for implant comparison as they eliminate confounding variables, such as bone quality, individual anatomy and ligamentous stability, and concomitantly allow standardized testing conditions.

Also, as outlined above, rotation displays to be the key motion of the atlanto-axial joint, whereas in this study the mobility was greatest in the sagittal plane. This can be most likely explained by the location of the pivot point ventrally of the dorsal spondylodesis, leading to a higher bending moment and consequently resulting in a greater leverage on the implant during flexion and extension.

Furthermore, this study focused on the comparison of the Harati technique to the Harms technique, both of which are dorsal stabilization techniques. Yet, as outlined above, indications for anterior stabilization are rare and mostly limited to younger patients with minor instability [4]. Therefore, this study investigated posterior techniques based on their more frequent implantation and wider indications. The Harms technique was used for comparison as it is one of the most stable dorsal stabilization techniques and well established in Germany.

Overall, this study was the first biomechanical study that was able to confirm the biomechanical superiority of the osteosynthesis technique described by Harati et al. over the one by Harms et al. Consequently, this novel technique can be regarded as a promising alternative for the treatment of atlanto-axial instabilities.

## Data Availability

All data are available upon request.
